# The Auxin Response Factor *OsARF25* Negatively Regulates Grain Size and Weight in Rice (*Oryza sativa* L.) by Activating the Expression of *SG1* and *OsOFP04*

**DOI:** 10.3390/plants14121808

**Published:** 2025-06-12

**Authors:** Xinrong Zhang, Yimeng Xu, Fengjun Xian, Shuya Liu, Jishuai Huang, Bin Xie, Jun Hu

**Affiliations:** State Key Laboratory of Hybrid Rice, Engineering Research Center for Plant Biotechnology and Germplasm Utilization of Ministry of Education, College of Life Sciences, Wuhan University, Wuhan 430072, China; 2022302041086@whu.edu.cn (X.Z.); 2022302041127@whu.edu.cn (Y.X.); 2019202040064@whu.edu.cn (F.X.); 2022202040080@whu.edu.cn (S.L.); huangjishuai28@whu.edu.cn (J.H.); 2020202040081@whu.edu.cn (B.X.)

**Keywords:** OsARF25, grain size, grain weight, BR signaling pathway, rice

## Abstract

Grain size and grain weight are critical factors influencing crop yield. In rice (*Oryza sativa* L.), the auxin response factor (OsARF) family proteins, key components of the auxin signaling pathway, function as transcription factors and play essential roles in regulating various plant growth and development processes, including seed development. Here, we identified that *Oryza sativa AUXIN RESPONSE FACTOR 25* (*OsARF25*) plays an essential role in regulating grain size and grain weight by activating the expression of *SHORT GRAIN 1* (*SG1*) and *Oryza sativa OVATE FAMILY PROTEIN 04* (*OsOFP04*). The *osarf25* mutants showed larger grains with increased grain length, grain width, and 1000-grain weight. Furthermore, molecular evidence demonstrated that OsARF25 functions as a transcriptional activator. RNA-seq analysis further identified its target genes *SG1* and *OsOFP04*. In addition, OsARF25 directly binds to the promoters of *SG1* and *OsOFP04* and activates their expression. Further, the *osarf25* mutant exhibited enhanced sensitivity to brassinolide treatment, confirming that the targeting of *SG1* and *OsOFP04* by OsARF25 mediates BR signaling. Taken together, our study revealed that *OsARF25* functions as a regulator of grain length, grain width, and grain weight by participating in the BR signaling pathway, and it has potential value for molecular breeding in rice.

## 1. Introduction

Rice (*Oryza sativa* L.) is one of the most important food crops in the world and the main food source for about half of the world’s population [1]. With the rapid growth of the global population and the deterioration of the environment resulting from climate change, agricultural production is facing great challenges [2]. Grain size, which is primarily determined by length, width, and thickness, is one of the key factors influencing grain weight and yield in crops [3]. Therefore, research on the mechanisms of the regulation of grain size and weight not only broadens the knowledge on the development of seeds but also helps breeders increase crop production by developing more high-yield and high-quality varieties.

Auxin, a key plant hormone, is essential for various processes of plant growth and development, including cell division, cell elongation, and cell differentiation [4]. The auxin signaling pathway initiates or mediates the developmental processes through auxin-regulated gene expression, which is normally involved in auxin response factors (ARFs) [5]. ARFs are transcription factors (TFs) that bind to the auxin-responsive elements (AuxREs) that contain a conserved motif, TGTCTC, and some variation of this motif, such as TGTCCC or TGTCAC, in the promoters of auxin-responsive genes and mediate auxin signal transduction [6]. A typical ARF protein contains three domains, a conserved N-terminal DNA-binding domain (DBD), a conserved C-terminal dimerization domain (CTD), and a non-conserved middle region (MR) [7]. The ARF DBD, classified as a plant-specific B3-type DNA-binding domain, recognizes and binds to AuxREs in target auxin response genes. This binding enables ARFs to either activate or repress transcription depending on the nature of their middle regulatory domain [5]. The CTDs are highly similar to domains III and IV of AUXIN/INDOLE-3-ACETIC ACID (Aux/IAA) proteins and promote the interaction between proteins through the homo- and heterodimerization of ARFs. Subsequently, the heterodimerization among ARF and AUX/IAA proteins achieves the regulation of auxin response genes [8]. The amino acid sequence length and composition of the MR determine the transcriptional regulatory activity (activation or repression) of the ARFs on the downstream gene. MR domains enriched in glutamine (Q), leucine (L), and serine (S) residues typically confer transcriptional activation, whereas those enriched in proline (P), serine(S), glycine (G), and leucine (L) residues are associated with repressive functions [9]. A previous study has revealed that there are 25 OsARF proteins in rice, and a bioinformatics analysis has predicted that nine of these proteins, including OsARF5, OsARF6, OsARF11, OsARF12, OsARF16, OsARF17, OsARF19, OsARF21, and OsARF25, act as transcriptional activators, while the remaining proteins function as transcriptional suppressors [9].

The ARFs play crucial roles in diverse aspects of plant growth and development, such as flower organogenesis and pattern [10], flower senescence and abscission [11], lateral root formation [12], epidermal cell and trichome formation [13], organogenesis [14], fruit maturation [15], biological and abiotic stress responses, and hormonal signaling pathways [16]. In rice, previous studies elucidated the molecular mechanisms by which OsARF proteins regulate grain size. OsARF6 binds to the AuxRE (TGTCAC) in the promoter of *OsAUX3*, a negative regulator of rice grain size, and alters auxin accumulation and distribution in glume cells, ultimately inhibiting their longitudinal elongation and consequently reducing both grain length and weight [17]. OsSK41 physically interacts with and phosphorylates OsARF4, which increases the accumulation of OsARF4, thereby negatively regulating rice grain size [18]. OsARF19 directly binds to the promoter of *OsSHI1* and activates its expression in response to auxin and BR. The *shi1* mutant exhibits typical brassinosteroid (BR)-deficient grain size, while plants overexpressing *OsARF19* display a BR-enhanced phenotype, such as lengthened grains [19]. OsIAA10 phosphorylated by TGW3 facilitates its interaction with OsTIR1 and its subsequent destabilization, which hinders its interaction with OsARF4 and then enhances the auxin signaling to regulate grain size. Plants overexpressing *OsIAA10* produce apparently longer grains, whereas *osiaa10* mutants, in contrast, exhibit obviously shorter seeds [20]. Moreover, our previous study reported that OsIAA3 interacts with OsARF16, which activates the expression of *Oryza sativa BRASSINOSTEROID UP-REGULATED 1-LIKE (OsBUL1)* to regulate grain size [21].

Brassinosteroids (BRs) are a group of plant steroidal hormones that regulate diverse biological processes, including leaf angle, plant height, stress responses, grain size, and weight [22]. Notably, rice plants that are deficient or insensitive to BRs produce shorter and smaller seeds, suggesting the critical role of BR signaling in determining grain yield [23,24,25]. Rice GSK3/SHAGGY-like kinase (GSK2) interacts with and phosphorylates DWARF AND LOW-TILLERING (DLT), a positive regulator that mediates several BR responses in rice, to carry out various BR responses, including the regulation of grain size [26]. qGL3 physically interacts with and dephosphorylates the GSK3/SHAGGY-like kinase 3 (OsGSK3) in rice, which modulates *Oryza sativa* BRASSINAZOLE RESISTANT1 (OsBZR1) phosphorylation and subcellular distribution to suppresses BR signaling to regulate the grain length [27]. *GRAINLENGTH2 (GL2)* encodes *Oryza sativa* GROWTH REGULATION FACTOR4 (OsGRF4), and OsGRF4 interacts with GSK2, which acts as the central negative regulator of rice BR signaling and inhibits its transcription activation activity of OsGRF4 to regulate grain length [28].

To further clarify the roles of ARFs in the development of rice grains, we focused on several OsARF transcriptional activators and generated their respective mutant lines. Phenotypic characterization revealed that the *osafr25* mutants produced significantly larger seeds compared to WT. Notably, transcriptional analysis revealed that *OsARF25* is highly expressed in the palea, lemma, and panicles related to seed development. Given the expression specificity and obvious grain phenotype, we aimed to investigate the molecular mechanism by which *OsARF25* regulates grain size in rice while other ARFs are currently under investigation in our lab.

In this study, we demonstrated that *OsARF25* negatively regulates grain length, grain width, and 1000-grain weight in rice, with its mutation significantly increasing the 1000-grain weight. In addition, we revealed that OsARF25 activates the expression of *SG1* and *OsOFP04* by directly binding to their promoters and participates in the BR signaling pathway through these two genes, thus regulating grain size and weight. Our findings advance the understanding of OsARF25-mediated regulatory mechanisms in rice grain development and expand the functional network of the ARF transcription factor family. Furthermore, a deeper understanding of the development of seeds provides potential directions to increase crop production.

## 2. Results

### 2.1. OsARF25 Is Highly Expressed in Palea, Lemma, and Panicles

**Figure 1:**
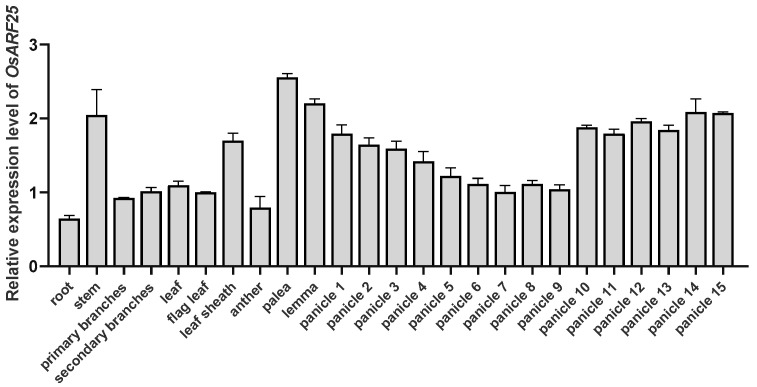
Expression analysis of *OsARF25*. RT-qPCR analysis of relative expression levels of *OsARF25* in different tissues from WT. The number after panicles indicates the length (cm); for example, panicle 1 indicates panicles 0~1 cm in length, panicle 2 indicates panicles 1~2 cm in length, and so on. Data are means (±SD) (*n* = 3). *OsActin* was used as an internal control for normalization.

To clarify the expression pattern of *OsARF25*, total RNA was isolated from root, stem, leaf, palea, lemma, branch, sheath, and panicles of different lengths in WT (Nipponbare) and used for RT-qPCR analysis. The results showed that *OsARF25* was widely expressed in both the vegetative and reproductive tissues, indicating that the *OsARF25* gene has a constitutive expression pattern. More specifically, the expression level of *OsARF25* was maintained at a high level in palea, lemma, and panicles. Meanwhile, a dynamic expression pattern was observed during panicle elongation (Figure 1). The expression levels of *OsARF25* initially declined in intermediate-length panicles (4~9 cm) but subsequently increased in panicles 10~15 cm in size. Taken together, these results suggested that *OsARF25* is constitutively expressed and exhibits relatively higher expression levels in palea, lemma, and panicles, implying its potential regulatory functions in panicle maturation and seed development.

### 2.2. OsARF25 Localizes into Nuclear and Functions as a Transcriptional Activator

**Figure 2:**
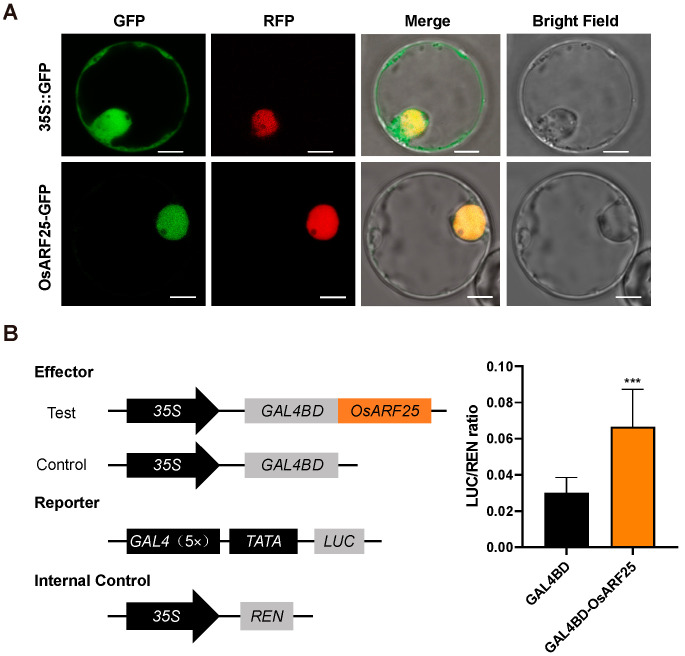
OsARF25 functions as a transcriptional activator. (**A**) Subcellular localization analysis of OsARF25. The scale bar is 5 μm. (**B**) OsARF25 process transcriptional activation in rice protoplasts. Data are means (±SD.) (*n* = 3); significant differences were determined using Student’s *t*-test: *** *p* < 0.001.

A previous study suggested that ARFs functioned as transcriptional factors. Firstly, to further investigate the subcellular localization of OsARF25, we fused OsARF25 to the N-terminus of the green fluorescent protein (GFP). GFP and OsARF25-GFP were transiently expressed in rice protoplasts, respectively. Meanwhile, a nuclear-specific marker D53 protein fused with RFP was selected as the nuclear marker. The results showed that the signals from OsARF25-GFP and D53-RFP were well co-localized in the nuclear (Figure 2A), demonstrating that OsARF25 is a nuclear-localized protein.

To further explore the transcriptional activity of OsARF25, we performed a dual-luciferase reporter assay in rice protoplasts. The full-length OsARF25 was fused in the frame with GAL4-BD to generate an effector. The effector, internal control, and reporter were co-expressed in rice protoplasts (Figure 2B). Compared with the control, a significantly increased LUC/REN ratio was detected, suggesting that OsARF25 has transcriptional activation activity (Figure 2B). In conclusion, these results demonstrate that OsARF25 functions as a transcriptional activator.

### 2.3. Bioinformatic Analysis of the OsARFs in Rice

**Figure 3:**
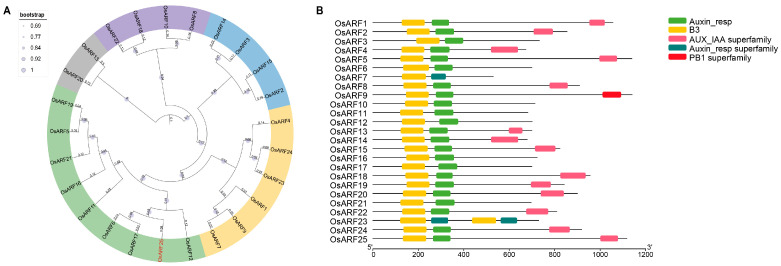
Bioinformatic analysis of the OsARF protein family in rice. (**A**) Phylogenetic tree of the OsARF protein family in rice. The tree was constructed using the adjacency method in the MEGA6 software. The bootstrap is set to 1000. (**B**) Conserved motif analysis of the OsARF proteins in rice.

To investigate the function of the ARF family in rice, a phylogenetic analysis based on the full-length protein sequences of OsARFs was carried out. The phylogenetic tree revealed that OsARF25 is evolutionarily related to OsARF6 [17] and OsARF12 [29], both of which have been reported to be involved in regulating grain size previously (Figure 3A). Then, the conserved motifs of the OsARF family were further analyzed, and the results showed that most members shared the B3 motifs, auxin response motifs, and Aux/IAA superfamily motifs (Figure 3B). Therefore, we speculated that OsARF25 might be involved in the auxin signaling pathway and play a potential role in grain size regulation.

### 2.4. The osarf25 Mutants Exhibited Increased Rice Grain Size and Weight

**Figure 4:**
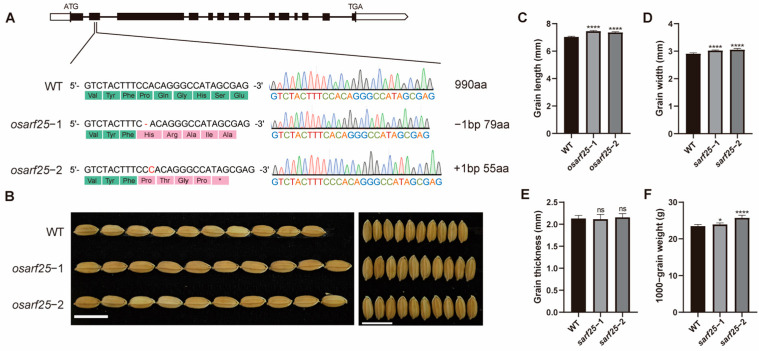
*OsARF25* negatively regulates grain size and grain weight. (**A**) Sequence analysis of the target sites in WT and *OsARF25* knockout lines (*osarf25*-1/-2). In *osarf25*-1 and *osarf25*-2 lines, a 1 bp deletion and 1 bp insertion in the second exon results in premature translation termination. (**B**) Grain phenotypes of WT and *osarf25* mutants shown in scale bars of 10 mm. (**C**–**F**) Phenotypic differences in grain traits between WT and *osarf25* mutants. Grain length (**C**), grain width (**D**), grain thickness (**E**), and 1000-grain weight (**F**) of *osarf25* mutants and WT were analyzed. Data are means (±SD.) (*n* = 10); significant differences were determined using Student’s *t*-test: * *p* < 0.05, **** *p* < 0.0001.

To gain insight into the function of OsARF25, we generated *OsARF25* knockout plants using CRISPR/Cas9 technology in a Nipponbare background. Two Cas9-free *osarf25* T_2_ homozygous mutants were obtained, named *osarf25-1* and *osarf25-2*, respectively. *osarf25-1* with 1 bp deletion and *osarf25-2* with 1 bp insertion were identified by Sanger sequencing, and both of them resulted in the premature termination of OsARF25 (Figure 4A). Meanwhile, we also generated transgenic plants overexpressing *OsARF25*, *OsARF25-OE-1*, and *OsARF25-OE-2*, driven by the ubiquitin promoter in a Nipponbare background. Subsequently, we focused on the grain size of both *osarf25* mutants and WT. The two knockout lines (*osarf25-1* and *osarf25-2*) exhibited significantly increased grain length, grain width, and grain weight compared to WT (Figure 4B). The average grain length in the lines *osarf25-1* and *osarf25-2* was about 7.45 mm and 7.36 mm, respectively, compared to 7.04 mm in WT plants (Figure 4C). The average grain width in the lines *osarf25-1* and *osarf25-2* was about 3.02 mm and 3.05 mm, respectively, compared to 2.91 mm in WT plants (Figure 4D). In addition, the 1000-grain weight was increased by 1.8% and 9.4% in the *osarf25-1* and *osarf25-2* lines (Figure 4F), respectively, compared to WT plants, but there was little change in grain thickness (Figure 4E). Further, the 1000-grain weight was decreased by 5.0% and 6.4% in *OsARF25-OE-1* and *OsARF25-OE-2* compared to WT plants (Figure S1). These results showed that the knockout of *OsARF25* increases rice grain length, grain width, and grain weight, suggesting that *OsARF25* negatively regulates grain size and grain weight in rice.

### 2.5. Transcriptome Analysis of the osarf25 Mutants by RNA-seq

**Figure 5:**
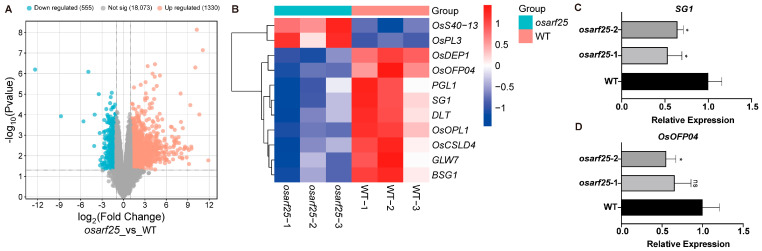
Screening of target gene for OsARF25. (**A**) Volcanic map of the differentially expressed genes. (**B**) Heat map of DEGs in regulating grain size. (**C**,**D**) RT-qPCR validation of *SG1* (**C**) and *OsOFP04* (**D**). Data are means (±SD.) (*n* = 3); significant differences were determined using Student’s *t*-test: * *p* < 0.05.

To elucidate the underlying mechanism of how *OsARF25* regulates the grain size and grain weight, RNA-seq analysis was carried out with the panicle RNAs from the *osarf25* mutants and WT. A total of 1885 differentially expressed genes (DEGs) in *osarf25* lines compared to the WT were found, among which 1330 were up-regulated genes and 555 were down-regulated genes (Figure 5A).

According to our above evidence that OsARF25 functions as a transcriptional activator, we first focused on the down-regulated genes. Considering its negative roles of regulating grain size and grain weight, we hypothesized that its downstream targets should be the negative regulators of grain size and grain weight. Subsequently, based on the above two rules, three candidate genes were selected from the DEGs dataset and further examined by RT-qPCR, among which *SG1* (LOC_Os09g28520) and *OsOFP04* (LOC_Os01g53160) were validated as candidate downstream genes (Figure S2). The results confirmed that both *SG1* and *OsOFP04* were down-regulated in *osarf25* mutants compared with WT (Figure 5C,D). Thus, *SG1* and *OsOFP04* were selected as the candidate downstream genes of OsARF25 for further validation.

### 2.6. OsARF25 Directly Binds to the SG1 and OsOFP04 Promoters

**Figure 6:**
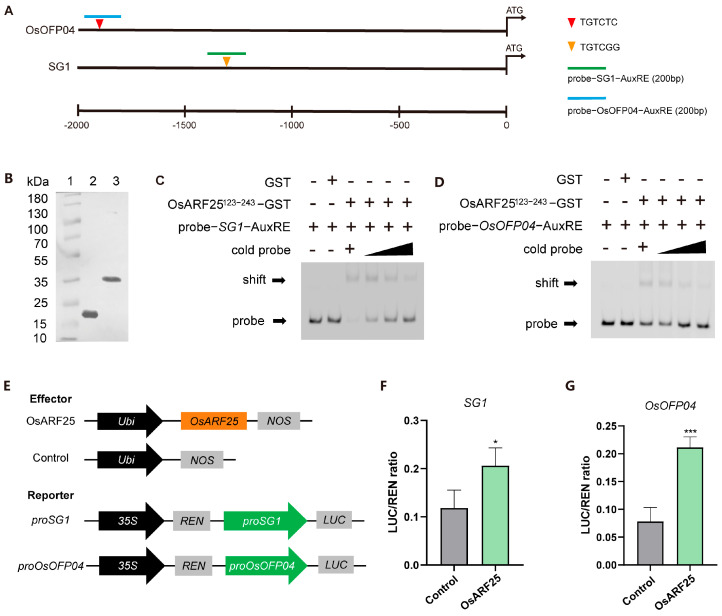
OsARF25 regulates *SG1* and *OsOFP04* by directly binding to their promoters. (**A**) Schematic diagram of predicted AuxREs in *proSG1* and *proOsOFP04*. (**B**) Western blotting of GST and recombinant OsARF25^123-243^-GST proteins. Lane 1: 180kDa Plus Prestained Protein Marker; Lane 2: GST protein; Lane 3: OsARF25^123-243^-GST. (**C**,**D**) An electrophoretic mobility shift assay (EMSA) of OsARF25^123-243^-GST binding to the *SG1* (**C**) and *OsOFP04* (**D**) promoters. OsARF25^123-243^-GST was incubated with the 6-FAM-labeled probe in the absence or presence of 2-fold, 5-fold, and 10-fold excesses of the corresponding competitors. (**E**–**G**) Dual-luciferase reporter assay of OsARF25 and the promoters of *SG1* (**F**) and *OsOFP04* (**G**) in rice protoplasts, containing the indicated constructs shown on the left (**E**). Data are means (±SD.) (*n* = 3); significant differences were determined using Student’s *t*-test: * *p* < 0.05, *** *p* < 0.001.

A previous study showed that ARFs regulate auxin signaling by binding to TGTCTC-containing AuxREs in the promoters of auxin-responsive genes [5,30]. To determine whether OsARF25 directly regulates the candidate genes *SG1* and *OsOFP04*, a bioinformatics analysis was performed to analyze the promoters of *SG1* and *OsOFP04*. Approximately 2 kb of promoter fragments from the translation start site (TSS) of *SG1* (*proSG1*) and *OsOFP04* (*proOsOFP04*) were analyzed by PlantCARE (https://bioinformatics.psb.ugent.be/webtools/plantcare/html/, accessed on 14 October 2024), respectively [31]. Several AuxREs were identified (Figure 6A), implying that *SG1* and *OsOFP04* may function as the downstream genes of OsARF25 in the auxin-signaling pathway. To confirm the direct binding activity of OsARF25, we expressed the full length of OsARF25; however, it failed to purify. Then, we expressed the B3 domain fused with a GST tag as the recombinant protein OsARF25^123-243^-GST according to a previous study [32]. Recombinant OsARF25^123-243^-GST was expressed and purified by an electrophoretic mobility shift assay (Figure 6B). The results revealed that recombinant OsARF25^123-243^-GST caused shift bands of the 6-FAM-labeled *proSG1* probe and *proOsOFP04* probe, respectively. Meanwhile, the shifted bands were markedly reduced with an increased non-labeled (cold) competitor (Figure 6C,D). Therefore, we concluded that OsARF25 directly binds to the AuxREs in *SG1* and *OsOFP04* promoters. Next, we performed transient expression assays to confirm the interaction in vivo. The *Ubi*::OsARF25 effector, *proSG1*::LUC reporter, and *proOsOFP04*::LUC reporter plasmids were respectively co-transformed into rice protoplasts. The co-expression of *Ubi*::OsARF25 and *proSG1*::LUC or *proOsOFP04*::LUC significantly increased the ratio of LUC/REN compared with the effects of the empty vector control (Figure 6E), solidly indicating transcriptional activation by OsARF25 on *SG1* and *OsOFP04*. All these results suggest that OsARF25 positively regulates the expression of *SG1* and *OsOFP04* by directly binding to their promoters.

## 3. Discussion

**Figure 7:**
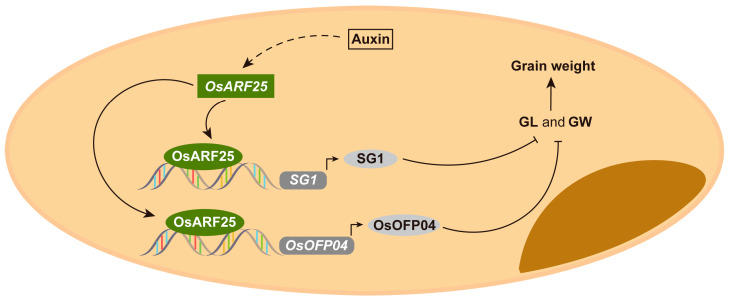
Proposed model explaining how OsARF25 acts upstream of *SG1* and *OsOFP04* to regulate grain size and grain weight in rice. OsARF25, a key component of auxin signaling, is thought to activate the expression of both *SG1* and *OsOFP04* by binding directly to their promoters. OsARF25 is suggested to negatively regulate grain size and grain weight by activating the expression of *SG1* and *OsOFP04*.

Grain size is one of the important factors affecting crop production. Rice yield is mainly determined by panicle numbers, grain numbers per panicle, and grain weight. Grain length, grain width, and grain thickness are the key components of rice grain size. It is reported that *SG1* negatively controls the grain length and *OsOFP04* negatively regulates the grain length and grain width [33,34]. In this study, we found that *OsARF25* was abundantly expressed in palea, lemma, and panicles. Moreover, the *osarf25* mutants exhibited typical BR-enhanced longer grains compared with WT (Figure 4). Further, genetic and molecular evidence has shown that OsARF25 acts as an upstream transcription activator of *SG1* and *OsOFP04* and can directly bind to the AuxREs in their promoters to activate their expression, which forms a novel regulation module on grain length and grain weight in rice through the BR signaling pathway (Figure 7).

In our study, we observed that the seeds of *OsARF25-OE* exhibited no significant differences in grain length, width, or thickness compared to those of WT. However, their thousand-grain weight was markedly lower than that of the WT. We speculate that this discrepancy may be attributed to variations in grain filling rate. Previous studies show that auxin also plays an important role in rice grain filling. *OsYUC11* mediates auxin biosynthesis in rice endosperm, which is essential for endosperm development [35]. *DEP1/qPE9–1* increases the content of auxin and cytokinin during the grain filling stage, which results in increased starch accumulation in rice [36]. At a high temperature, OsIAA29 enhances the transcriptional activation activity of OsARF17 to regulate the grain filling [37].

BR is a special phytosterol hormone that plays an important role in plant growth and development including, plant height, leaf angle, and seed formation [18]. ARF is a family of transcription factors, normally regulating many auxin-responsive genes and mediating responses to the plant hormone auxin [6]. In previous studies, ARFs have been reported to target the AuxRE to mediate the expression of auxin-responsive genes and BR-responsive genes. In rice, OsARF19 directly targets the promoter of the BR receptor kinase gene, *OsBRI1*, thereby modulating BR perception levels to control leaf angle regulation [38]. OsIAA1 and OsARF1 are one of the matched interaction pairs of the Aux/IAA and ARF families that are involved in BR hormone responses and plant morphogenesis [39]. *OsARF4* regulates leaf inclination via auxin and BR pathways in rice [40]. DS1/OsEMF1 interacts with OsARF11 to control rice architecture by regulating BR signaling [41]. Despite abundant advances having been made in understanding the link between ARFs and the BR signaling pathway in recent years, the mechanism by which ARFs regulate rice grain size through BR signaling is largely unknown. In our study, we found that *osarf25* showed increased sensitivity to BR through the lamina joint inclination assay and coleoptile elongation assay (Figure S3). *OsOFP04* has been shown to be an inhibitor of BR synthesis and signal transduction [34]. *SG1* decreases cellular proliferation to decrease the response to BRs and elongation of organs [31]. Therefore, we draw the conclusion that OsARF25 binds to the promoter of *OsOFP04* and *SG1* and activates their expression to regulate the grain size and grain weight through the BR signaling pathway. This study improves the regulatory network in which ARFs participated in the regulation of grain size and advances our understanding of hormonal crosstalk in grain development.

Previously, several studies identified the upstream regulatory genes of *OsARF25*. OsmiR167a represses its targets, *OsARF12*, *OsARF17*, and *OsARF25*, to control the rice tiller angle by fine-tuning auxin asymmetric distribution in the shoots [42]. OsmiR5488 takes part in the regulation of the seed-setting rate and down-regulates the targeted gene *OsARF25* [43]. *OsTIR1* mediates the sugar import into rice endosperm via enhancing the expression level of *OsARF25*, which interacts with sugar transporter *OsSWEET11* [44]. OsNAC2 regulates the expression of *OsARF25, GH3.6*, *GH3.8*, and *OsCKX4* to modulate rice root development [45]. Here, we identified the downstream genes, *SG1* and *OsOFP04*, of *OsARF25* involved in regulating rice grain size, but the upstream regulators of this pathway remain unclear. Thus, elucidating the upstream regulatory factors of *OsARF25* is quite important, which might have great application potential for yield improvement in rice.

## 4. Materials and Methods

### 4.1. Bioinformatic Analysis

A total of 25 OsARFs are listed in Table S1. The corresponding protein sequences were used to construct the phylogenetic tree by using the MEGA6 software with the neighbor-joining method [46], and the bootstrap was set to 1000. The constructed phylogenetic tree was then further refined by ITOL (https://itol.embl.de/, accessed on 18 March 2025). The conserved motifs of the ARF family were analyzed using the NCBI website (https://www.ncbi.nlm.nih.gov/Structure/cdd/, accessed on 18 March 2025) [47].

### 4.2. Plant Materials and Growth Conditions

The rice materials used in this study were the *osarf25* mutants, *OsARF25-OE* lines, and their corresponding wild-type variety, Nipponbare (WT). All plants were grown in the field of Wuhan University (Wuhan, Hubei Province, China) under proper management.

### 4.3. Plasmid Construction and Transformation

The coding sequences (CDS) without a stop codon of *OsARF25* (2697 bp) were amplified from the WT and inserted into the binary vector pCAMBIA1301 driven by the ubiquitin promoter. The Flag tag was incorporated into the C-terminal of *OsARF25* to generate *OsARF25-OE.* The CDS was inserted into the vector using the ClonExpress II One-Step Cloning Kit (Vazyme, Nanjing, China). The recombinant vector was then introduced into the *Agrobacterium tumefaciens* strain EHA105, which was used to transform the Nipponbare cultivar. The *osarf25* mutant was purchased from BIOGLE (Changzhou, Jiangsu, China). The primers used are listed in Table S2.

### 4.4. Total RNA Extraction and Expression Analysis

Total RNA was extracted from different plant tissues using Trizol reagent (Invitrogen, Waltham, MA, USA). The cDNA was synthesized using Hifair^®^ III 1st Strand cDNA Synthesis SuperMix for qPCR (gDNA digester plus) (YEASEN, Shanghai, China) and used as the template for RT-qPCR analysis. RT-qPCR assays were performed with CFX96 Touch Real-Time PCR Detection System using the Hieff^®^ qPCR SYBR Green Master Mix (No Rox) (YEASEN, Shanghai, China) according to the manufacturer’s recommended protocol. Each experiment was conducted with three biological replicates.

### 4.5. Subcellular Localization Analysis

The full length of *OsARF25* was cloned into vector HBT-sGFP driven by the *Cauliflower mosaic virus (CaMV) 35S promoter.* Stems of 10-day-old etiolated seedlings (WT) were cut into 0.5 mm strips and balanced by 0.6 M Mannitol for 10 min. The strips were placed in 20 mL of enzyme solution (0.6 M Mannitol, 10 mM MES, 1.5% cellulase R-10, 0.75% macerozyme R-10, 0.1% BSA, 10 mM CaCl_2_, 20 μg/mL ampicillin, 0.4 μL/mL 2-Mercaptoethanol) for 4–5 h at 28 °C with shaking at 80 rpm. An amount of 20 mL of W5 solution (154 mM NaCl, 125 mM CaCl_2_, 5 mM KCl, 5 mM glucose, 2 mM MES) was added to release the protoplasts. After filtering through 35 μm nylon mesh, protoplasts were collected by centrifuging at 450× *g* for 3 min. The collected protoplasts were resuspended in an appropriate volume of MMG solution (15 mM MgCl_2_, 4 mM MES, 0.6 M Mannitol). 35S::OsARF25-GFP and the nuclear localization marker D53-RFP, at about 8 μg, respectively, were mixed with 100 μL protoplasts (usually 2 × 10^4^ cells/mL). An amount of 120 μL 40% PEG solution (0.2 M Mannitol, 0.1 M CaCl_2_, 4 g/mL PEG4000) was added into the DNA and protoplast mixture, immediately mixed and gently shaken, and then the mixture was incubated for 30 min at 28 °C. After incubation, 500 μL of W5 solution was added to terminate the reaction. The protoplasts were then collected by centrifuging at 450× *g* for 3 min, resuspended in 1 mL of W5 solution, and incubated for 16–18 h at 28 °C in the dark. Images were collected using a high-resolution confocal microscope (TCS SP8; Leica, Wetzlar, Germany).

### 4.6. Dual-Luciferase Reporter Assay

A total of 2 kb of the promoter sequences of candidate downstream genes were cloned into the *pGREEN II 0800-Luc* vector to create the promoter–reporter-driven firefly luciferase constructs *pGreen0800II-proSG1::LUC* and *pGreen0800II-proOsOFP04::LUC*. The *OsARF25* CDS was cloned into the *pCAMBIA1301* vector to create *pCAMBIA1301-OsARF25*, which was used as the effector vector. Both vectors were co-expressed in rice protoplasts (WT). Co-transfected protoplasts were cultured for 16 h in the dark. Luciferase activities were measured using the Dual-Luciferase^®^Reporter (DLR™) Assay System (Promega, WI, USA). The relative luciferase activity was calculated as the ratio of firefly luciferase to Renilla luciferase (LUC/REN). The primers used are listed in Table S2.

### 4.7. Transcriptional Activation Analysis

To conduct an analysis of the transcriptional activation of OsARF25 within rice protoplasts, the CDS of OsARF25 was inserted into the GAL4 DNA-binding domain (DBD) vector, leading to the generation of the GAL4BD-OsARF25 construct. The luciferase gene, which incorporated five copies of the binding sites for GAL4, was employed as a reporter gene. Moreover, the Renilla luciferase gene, which was driven by the CaMV 35S promoter, was utilized as an internal control. Both vectors were co-expressed in rice protoplasts (WT). Co-transfected protoplasts were cultured for 16 h in the dark. Luciferase activities were measured using the Dual-Luciferase^®^Reporter (DLR™) Assay System (Promega, WI, USA). The relative luciferase activity was calculated as the ratio of firefly luciferase to Renilla luciferase (LUC/REN). The primers used are listed in Table S2.

### 4.8. RNA Sequencing Analysis

Total RNA samples were prepared from young panicles of WT and the *osarf25*-1 mutant with three biological replicates. Total RNA was extracted and sequenced by SeqHealth (Wuhan, Hubei, China). All DEGs between the WT and *osarf25* plants are listed in Table S3.

### 4.9. Western Blotting

The coding sequence of the B3 domain (123–243 aa) of OsARF25 was cloned into the pGEX-6P-1 vector [30]. The recombinant protein OsARF25^123-243^-GST was expressed in the Escherichia coli Rosetta (DE3) strain and purified using GSTSep Glutathione MagBeads (Yeasen, Shanghai, China) according to the manufacturer’s protocol. The recombinant protein OsARF25^123-243^-GST and GST were separated by sodium dodecyl sulfate polyacrylamide gel electrophoresis (SDS-PAGE) and transferred to a polyvinylidene fluoride (PVDF) membrane that was used for subsequent Western blotting analysis according to standard procedures. Rabbit anti-GST-tag monoclonal first antibody (ABclonal, AE007) was used for the detection of OsARF25^123-243^-GST protein.

### 4.10. Electrophoretic Mobility Shift Assay

6-FAM-labeled and unlabeled primers were synthesized by GenScript (Nanjing, Jiangsu, China). The binding reaction system (20 μL) was assembled with the following components: 50 ng DNA probe, 2 μg GST-tagged OsARF25^aa123-243^ recombinant protein, 2 μL 10× binding buffer (100 mM Tris, 500 mM KCl, 10 mM DTT, pH 7.5), and supplemented with 1 μL 1 M KCl, 1 μL 50% (*v*/*v*) glycerol, 1 μL 0.5 M EDTA, 1 μL 1% (*v*/*v*) Nonidet P-40, and 1 μL 1 mg/mL poly(dI-dC). Following 20 min incubation at 25 °C, reaction products were resolved by 5% native acrylamide gel electrophoresis and visualized using a Typhoon Trio Imager (GE, Cytiva, Shanghai, China). The primers used are listed in Table S2.

### 4.11. Lamina Joint Inclination Assay

The second leaf (including leaf and sheath) was excised from the two-week-old seedlings of the WT and *osarf25*. The excised leaves were immersed in 1 μM 24-epibrassinolide(24-EBL) or a control (ethanol absolute) and cultivated for 36 h in darkness at 28 °C. Ten samples were used for each test. ImageJ 1.54p software was used to evaluate the leaf angles.

### 4.12. Coleoptile Elongation Assay

Fifteen seeds of WT or *osarf25* were sterilized and grown on 0.3% agar medium with 1 μM 24-EBL or ethanol absolute under continuous dark at 28 °C for 5 days. The length of the coleoptiles was measured and the results were statistically analyzed. The increase in the length of coleoptiles was calculated.

## 5. Conclusions

In conclusion, this study revealed that *OsARF25* functions as a negative regulator of grain length, grain width, and grain weight by activating the expression of grain-size-regulated genes, *SG1* and *OsOFP04*, which also participate in the BR signaling pathway. Meanwhile, the *osarf25* mutants showed a phenotype of increased grain length, grain width, and grain weight. To investigate the mechanism, we first performed expression analysis, which revealed that the expression level of *OsARF25* was particularly high in palea, lemma, and panicles. Then, the results of the subcellular localization assay and dual-luciferase reporter assay showed that the OsARF25 protein acts as a transcriptional activator. The candidate genes of OsARF25 were screened by RNA-seq and verified by RT-qPCR assays. Both *SG1* and *OsOFP04* were involved in the BR signaling pathway as the downstream genes of OsARF25. Furthermore, lamina joint inclination and coleoptile elongation assays were performed to confirm that OsARF25 was involved in the BR signaling pathway. Taken together, our study elucidates both the spatiotemporal expression profile and biological role of *OsARF25* in rice, while systematically uncovering its dual regulatory capacity in coordinating auxin and BR signaling pathways. These findings establish *OsARF25* as a pivotal genetic determinant for molecular breeding strategies targeting grain yield.

## Data Availability

All data supporting the findings of this study are available within the paper and Supplementary Materials.

## Supplementary Materials

The following supporting information can be downloaded at: https://www.mdpi.com/article/10.3390/plants14121808/s1. Figure S1: Grain phenotypes of WT and *OsARF25-OE* plants; Figure S2: RT-qPCR validation of *DLT*; Figure S3: The sensitive test of WT and *osarf25* mutants to exogenous BR treatments; Table S1: Gene IDs of 25 members of OsARF family in rice; Table S2: Primers used in this study; Table S3: DEGs between the WT and *osarf25* plants.
