# Supplementary material for: The Auxin Response Factor OsARF25 Negatively Regulates Grain Size and Weight in Rice (Oryza sativa L.) by Activating the Expression of SG1 and OsOFP04
Source: Plants (Basel). 2025 Jun 12;14(12):1808. doi: 10.3390/plants14121808 (PMC12196904; doi:10.3390/plants14121808)
Supplement: Supplementary file 1 [file plants-14-01808-s001.zip › plants-3643319-supplementary/Supplementary Figure0429.pdf]

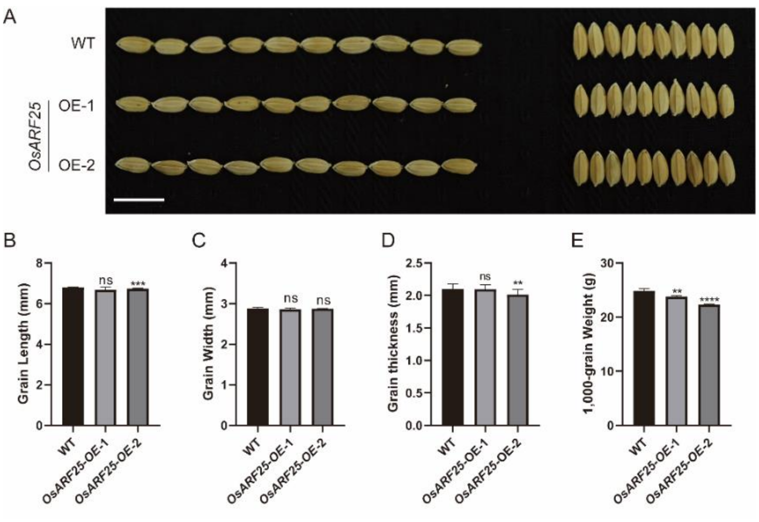


**Figure S1.** Grain phenotypes of wild type (WT)and *OsARF25-OE* plants.

(**A**) Grain phenotypes of WT and *OsARF25*-OE lines, shown in scale bar of 10 mm. (**B-E**) Phenotypic differences in grain traits between WT and *OsARF25*-OE lines. Grain length (**B**), grain width (**C**), grain thickness (**D**), and 1,000-grain weight (**E**) of *OsARF25-OE* lines and WT were analyzed. Data are means (± SD.) ($n$ = 5), significant differences were determined using Student's $t$-test: *$P$ < 0.5, **$P$ < 0.01, ****$P$ < 0.0001.

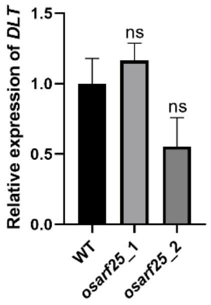


**Figure S2.** RT-qPCR validation of *DLT*. Data are means (± SD.) ($n$ = 3), significant differences were determined using Student's $t$-test.

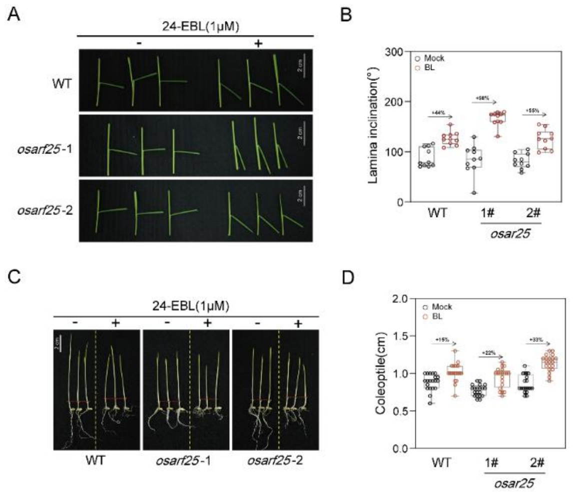

**Figure S3.** The sensitive experiments of WT and *osarf25* mutants to exogenous BR treatments. (**A**) Lamina inclination of WT, *osarf25*-1, and *osarf25*-2 seedlings with or without 24-epibrassinolide (24-EBL) treatment. (**B**) Comparisons of lamina inclination between WT, *osarf25*-1, and *osarf25*-2 as exemplified in (A) (*n* = 10). (**C**) Coleoptile elongation of Nip, *osarf25*-1, and *osarf25*-2 seedlings with or without 24-eBL treatment. Red hyphens indicate the tops of coleoptiles. (**D**) Comparisons of coleoptile elongation of Nip, *osarf25*-1, and *osarf25*-2 in response to 24-EBL treatment (*n* = 15).
